# [Retracted] MicroRNA‑320a suppresses tumour cell proliferation and invasion of renal cancer cells by targeting FoxM1

**DOI:** 10.3892/or.2024.8754

**Published:** 2024-06-10

**Authors:** Shiyue Zhao, Yangwei Wang, Yan Lou, Yonggang Wang, Jing Sun, Manyu Luo, Wen Li, Lining Miao

Oncol Rep 40: 1917–1926, 2018; DOI: 10.3892/or.2018.6597

Subsequently to the publication of the above paper, the authors drew to the attention of the Editorial Office that they made a couple of errors in terms of the data assembly in Figs. 2 and 4 in their paper; specifically, the Transwell assay data shown for the ‘miR-320a+/FoxM1+’ panel in Fig. 5D on p. 1923 also appeared as the ‘ACTN/NC’ data panel in Fig. 4E on the same page (Fig. 4E contained the erroneously duplicated panel). In addition, data featured in Fig. 2D of the above paper were strikingly similar to data that appeared in Fig. 6e of the following paper, published subsequently to this article, written by different authors (although a Dr Shiyue Zhao worked in the molecular biology laboratory of Harbin Medical University from 2017 to 2018, and the research collaboration was conducted with Dr Chenlong Li's research group): Li C, Zheng H, Hou W, Bao H, Xiong J, Che W, Gu Y, Sun H and Liang P: Long non-coding RNA linc00645 promotes TGF-β-induced epithelial-mesenchymal transition by regulating miR-205-3p-ZEB1 axis in glioma. Cell Death Dis 10: 17, 2019. Finally, after having conducted an independent investigation of the data in this paper, the Editorial Office noted that one of the Petri dish images in Fig. 2C was also strikingly similar to data that appeared in Fig. 2H of the abovementioned article in the journal *Cell Death & Disease*.

After having considered the authors' request for corrigendum, in view of the problems that were identified with the data, the Editor of *Oncology Reports* has decided that, owing to a lack of confidence in the presented data, the paper should instead be retracted from the journal. After having informed the authors of this decision, they accepted the decision to retract this paper. The Editor apologizes to the readership for any inconvenience caused.

